# Genetic correction of concurrent α- and β-thalassemia patient-derived pluripotent stem cells by the CRISPR-Cas9 technology

**DOI:** 10.1186/s13287-022-02768-5

**Published:** 2022-03-07

**Authors:** Lingli Li, Hongyan Yi, Zheng Liu, Ping Long, Tao Pan, Yuanhua Huang, Yongsheng Li, Qi Li, Yanlin Ma

**Affiliations:** 1 Hainan Provincial Key Laboratory for Human Reproductive Medicine and Genetic Research, Reproductive Medical Center, International Technology Cooperation Base “China-Myanmar Joint Research Center for Prevention and Treatment of Regional Major Disease” By the Ministry of Science and Technology of China, The First Affiliated Hospital of Hainan Medical University, Hainan Medical University, 3 Longhua Road, Haikou, 570102 Hainan China; 2 Key Laboratory of Tropical Translational Medicine of Ministry of Education, Hainan Medical University, Haikou, Hainan China; 3 Hainan Provincial Clinical Research Center for Thalassemia, The First Affiliated Hospital of Hainan Medical University, Hainan Medical University, Haikou, Hainan China; 4 College of Medical Laboratory Science, Guilin Medical University, Guilin, Guangxi China; 5 College of Biomedical Information and Engineering, Hainan Medical University, Haikou, 571199 China

**Keywords:** Thalassemia, CRISPR-Cas9 technology, Human-induced pluripotent stem cells, HBB gene, HBA2 gene

## Abstract

**Background:**

Thalassemia is a genetic blood disorder characterized by decreased hemoglobin production. Severe anemia can damage organs and severe threat to life safety. Allogeneic transplantation of bone marrow-derived hematopoietic stem cell (HSCs) at present represents a promising therapeutic approach for thalassemia. However, immune rejection and lack of HLA-matched donors limited its clinical application. In recent years, human-induced pluripotent stem cells (hiPSCs) technology offers prospects for autologous cell-based therapy since it could avoid the immunological problems mentioned above.

**Methods:**

In the present study, we established a new hiPSCs line derived from amniotic cells of a fetus with a homozygous β41-42 (TCTT) deletion mutation in the HBB gene and a heterozygous Westmead mutation (*C* > *G*) in the HBA2 gene. We designed a CRISPR-Cas9 to target these casual mutations and corrected them. Gene-corrected off-target analysis was performed by whole-exome capture sequencing. The corrected hiPSCs were analyzed by teratoma formation and erythroblasts differentiation assays.

**Results:**

These mutations were corrected with linearized donor DNA through CRISPR/Cas9-mediated homology-directed repair. Corrections of hiPSCs were validated by sequences. The corrected hiPSCs retain normal pluripotency. Moreover, they could be differentiated into hematopoietic progenitors, which proves that they maintain the multilineage differentiation potential.

**Conclusions:**

We designed sgRNAs and demonstrated that these sgRNAs facilitating the CRISPR-Cas9 genomic editing system could be applied to correct concurrent α- and β-thalassemia in patient-derived hiPSCs. In the future, these corrected hiPSCs can be applied for autologous transplantation in patients with concurrent α- and β-thalassemia.

## Introduction

Thalassemia is an inherited autosomal recessive blood disorder prevalent in the Mediterranean, Middle East, Indian subcontinent and Southeast Asia [1, 2]. Thalassemia has been classified into three main types, which include α-thalassemia, β-thalassemia, and concurrent α- and β-thalassemia [3]. In Southern China, α-thalassemia and β-thalassemia are the main two types observed [4]. It was reported that the most commonly detected mutation for α-thalassemia was SEA/αα (31.53%); the most common mutation for β-thalassemia was CD41/42 (30.27%) in Hainan province, southernmost China [5]. The clinical phenotypes of concurrent α- and β-thalassemia depend on how many of the four genes for α globin or two genes for β globin are missing [6]. Genetics analysis revealed that α-thalassemia is usually due to the deletion mutations within the α-globin gene, while most cases of β-thalassemia are caused by nondeletional mutations [7]. Based on clinical and laboratory reports, αWSα is the most common nondeletional α-thalassemia in Southern China. In Southeast Asia, the 4-bp deletion (-CTTT) at codon 41/42 (CD41/42) of the human β-globin gene represents the most common β-thalassemia mutations [8].

Patients suffering from severe anemia caused by thalassemia need lifelong blood transfusion [9]. At present, allogeneic HSC transplantation is the only practically available option with a high curative rate [10]. However, the outcome of HSC transplantation is strongly influenced by factors such as immune rejection, histocompatibility and the source of stem cells [11, 12]. Alternatively, researchers are pinning their hopes on gene therapy. Gene therapy is the process of replacing defective genes with healthy ones to help the body fight or treat disease [13]. As one of the gene therapy methods, the CRISPR technology modifies DNA with greater precision than existing technologies [14]. An advantage the CRISPR-Cas9 system offers over other mutagenic techniques, like zinc-finger nucleases and transcription activator-like effector nucleases, is its relative simplicity and versatility [15]. In the present study, we recruited a thalassemia patient with β41-42 (TCTT) deletions in the human β-globin (HBB) gene and a Hb-WS mutation (ααWS/αα) in the human hemoglobin alpha 2 (HBA2) gene. The human-induced pluripotent stem cells (hiPSCs) were derived from the patient’s amniotic cells and the CRISPR technology was applied to correct these two mutations. Finally, the gene-corrected hiPSCs retained normal pluripotency. It could be differentiated into hematopoietic progenitors by performing a directed differentiation assay in vitro. It proved that they maintained the multilineage differentiation potential.

## Methods

### Cell culture and hiPSC generation

The amniotic fluid used in this study was donated by a couple carrying thalassemia mutations. They have conducted amniocentesis for prenatal diagnosis in our department. The couple signed written informed consent for donating amniotic fluid. After the genetic testing, the remaining amniotic fluid was used to generate the hiPSC, following the procedures described previously [16]. Briefly, when the amniotic fluid cells reached the confluence of 70%-85%, the cells were digested with 0.25% trypsin and 1.2 × 10^6^ cells were resuspended with 100 μl DPBS; then, 6 μg plasmid PEP4-E02S-ET2K (Addgene number 20927) and 4 μg PCEP4-miR302-367 (including miR302a, b, c, d and miR 367) were added and mixed gently. The electroporation was conducted under the 200 V volts for 200 us. The electroporated cells were then cultured in Chang Amnio medium. When 40% confluence has occurred, the Chang Amnio medium was replaced with the induced medium. Then ES-like colonies were picked up; the induced hiPSCs were cultured in a matrigel-coated 6-well plate and maintained in mTesR1 culture medium (StemCell Technologies, Canada).

### CRISPR-Cas9 and donor vectors construction

HBB-sgRNA and HBB-donor were used to correct the mutations in HBB. Both gRNAs targeting HBB and HBA2 were designed by the CRISPR online tool (http://crispr.mit.edu/). The complementary annealed sgRNA oligonucleotides were inserted into the vector PX330 vector obtained from Addgene (Cambridge, USA). The primers were incubated at 16 °C for 2 h. After annealing, the products were transformed into DH5-alpha competent cells. Five monoclonal bacteria in LB medium containing 100 μg/ml benzylamine with temperature resistance at 37 °C were picked at random and centrifuged at 200 r/min for 16 h. The plasmid was extracted after the positive cloning sequence was identified.

**Fig. 1:**
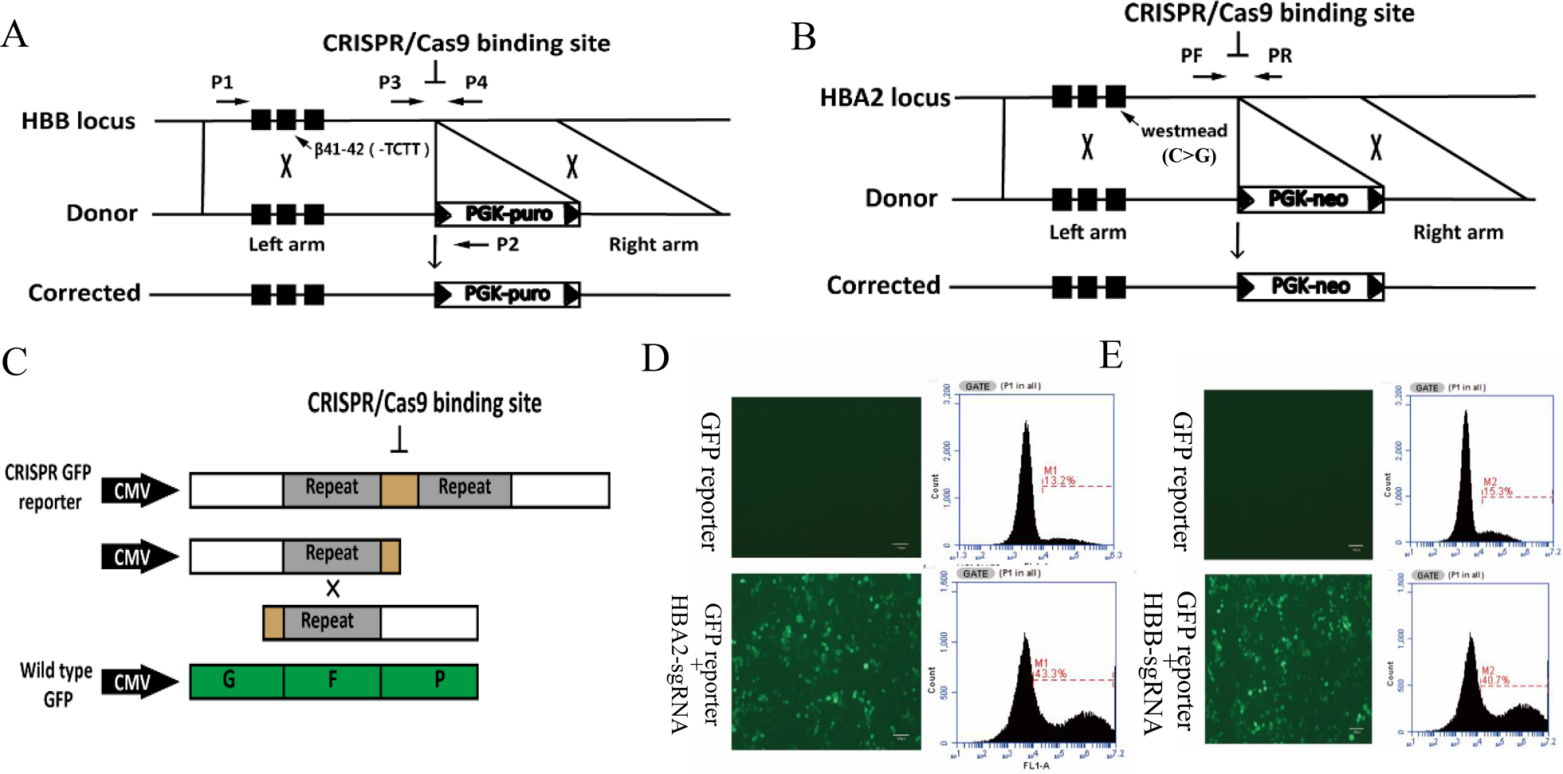
Strategy for correction of the thalassemia mutations and evaluation of the cleavage activity of gRNAs. **A** Schematic of the gRNAs for targeting the β-41/42 deletion mutant in the HBB gene. The oligos for β-sgRNA were designed in 600 bp downstream of the last exon of HBB. The primers used in this construct are AEXON-F/BEXON-R. **B** Schematic of the gRNAs for targeting the Westmead point mutation in the HBA2 gene. The oligos for α2-sgRNA were designed in 200 bp downstream of the last exon of HBA2. The primers used in this construct are HBA2 mut-F/R. **C** The complementary annealed sgRNA oligonucleotides were inserted into vector PX330. The sgRNA and PAM (NGG) sequence was inserted into the middle of the GFP gene and introduced into pTP53-GFP-reporter. After the targeting DNA was cut by Cas9, homologous recombination of the duplications occurred, resulting in the formation of a full-length GFP. **D** GFP signals were significantly increased, demonstrating the efficient cleavage activity of HBA2-sgRNA. **E** GFP signals were significantly increased, demonstrating the efficient cleavage activity of HBB-sgRNA

The primers HBBL-F/R and HBBR-F/R were used to amplify the left and right arm of HBB donor from the wild-type genome, respectively. The left arm, 2.3 kb, covered the whole HBB gene, and the right arm length had 1.5 kb, both of which were inserted in the Psimple-18 T vector and flanked the PGK-puromycin cassette (Fig. 1A). Primers sequences are listed in Table 1. The primers HBA2 L-F/R amplified the 800-bp left homology arm, and HBA2 R-F/R amplified the 700-bp right homology arm. The two arms were amplified from the wild-type genomic DNA and inserted into the pUC-57 vector. A lox P-flanked PGK-neomycin cassette was inserted between the two arms (Fig. 1B). Primers sequences are listed in Table 1.

**Table 1:** Primers used in this study

Primer	Sequence
HBB-LA-F	CCGAAGCTTGAATTCCTCGAGGCGGCCGCAGTGCCAGAAGAGCCAA
HBB-LA-R	AATCCCGGGGAATTCGTCGACATAACTTCGTATAGCATACAT
HBB-RA-F	CCGAAGCTTGAATTCCTCGAGATAACTTCGTATAATGTATGC
HBB-RA-R	AATCCCGGGGAATTCGTCGACGCGGCCGCGGTATACCTTGTGAAAT
HBB-sgRNA-F	CGAGATGGTTTCTCCTCGCCTGG
HBB-sgRNA-R	GGCGAGGAGAAACCATCTCG
P1	GTAGCAATTTGTACTGATGGTATGGGGC
P2	GGTGGATGTGGAATGTGTGCGAGG
P3	CAGCCTTAGTTGTCTCTGTTGTCTTA
P4	GGTGGTTGATGGTAACACTATGCTA
AEXON-F	CAATCTACTCCCAGGAGCAGGGA
BEXON-R	CGTCTGTTTCCCATTCTAAACTGTACC
HBB-GFP-sgRNA-F	GATCCGAGATGGTTTCTCCTCGCCTGG
HBB-GPF-sgRNA-R	AGCTCCAGGCGAGGAGAAACCATCTCG
HBA2-sgRNA-F	CACCGATGGAGAGCGTATGTTAAC
HBA2-sgRNA-R	AAACGTTAACATACGCTCTCCATC
HBA2-GFP-sgRNA-F	GATCGATGGAGAGCGTATGTTAACTGG
HBA2-GFP-sgRNA-R	AGCTCCAGTTAACATACGCTCTCCATC
HBA2-LA-F-EcoR1	CGGAATTCTCCTGCCGACAAGACCAAC
HBA2-LA-R-Cla1	CCATCGATCTCCATTGTTGGCACATTCC
HBA2-RA-F-Sal1	ACGCGTCGACAGGCAGTGGCTCAGAAGC
HBA2-RA-R-BamH1	CGGGATCCCTGTGAGGCGCAGGAAGA
PR	ACCGTGCTGACCTCCAAATAC
PF	ACTCCAGCCACCTACCCT
HBA2-Seq-R	ACTGACCCTCTTCTCTGCAC
HBA2-Seq-F	TGCCCACTCAGACTTTATTCAA
OCT4-F	CCTCACTTCACTGCACTGTA
OCT4-R	CAGGTTTTCTTTCCCTAGCT
Nanog-F	TGAACCTCAGCTACAAACAG
Nanog-R	TGGTGGTAGGAAGAGTAAAG
SOX2-F	CCCAGCAGACTTCACATGT
SOX2-R	CCTCCCATTTCCCTCGTTTT
GATA4-F	CAGAAAACGGAAGCCCAA
GATA4-R	TTGCTGGAGTTGCTGGAAG
T-F	GTGGGCCTGGAGGAGAGCGA
T-R	TTGTCCGCCGCCACGAAGTC
PAX6-F	TTGCTTGGGAAATCCGAG
PAX6-R	TGCCCGTTCAACATCCTT

*F* Forward; *R* reverse

### Green fluorescent protein (GFP) reporter assay

The designed sgRNA sequence was inserted between two repeating coding sequences with 205 bp, and then, the compound sequence was inserted into the pT53 plasmid (Fig. 1C). When sgRNA plasmid and GFP reporter were co-transfected into 293 T cells, the sgRNA cleaves the sgRNA sequence of GFP reporter through homologous recombination of repeating sequence. Finally, a complete GFP sequence was generated, thus expressing GFP, proving the validity and activity of sgRNA designed. Briefly, to test the sgRNA activity, GFP reporter and sgRNA plasmids were co-transfected into 293 T cells by calcium phosphate precipitation. Briefly, 293 T cells were cultured in 12-well plates with 5 × 10^5^ cells per well on the day before transfection. 24 h after transfection, the medium was replaced with 750 µL fresh 293 T medium and 250 µL mixture that contained plasmids, CaCl_2_, HEPES-buffered saline and ddH_2_O. The plasmids included 1 µg GFP reporter and 1 μg sgRNA plasmids. The medium was replaced after 12 h, and a picture was taken under fluorescent microscopy (Olympus, X71, Japan) after 48 h. The cells were suspended in 800 µL of PBS for flow cytometry (BD FACS Arial II, US) analysis. The flow cytometry data were analyzed by C6 Channel.

### Electroporation and drug selection

To correct the HBB β41-42/β41-42 mutations, 2 µg of HBB donor DNA and 4 µg of HBB-sgRNA plasmids were transfected into 1 × 10^6^ hiPSCs through electroporation. The cells were then cultured in Matrigel-coated 6-well plates with Y-27632 (10 nM, Sigma) for 1 day. Puromycin (0.5 µg/mL) was added into the mTeSR medium 3 days after the cell confluency reached 60%, and then, the concentration of puromycin was changed to 1 µg/mL and lasted for 4 days. The positive clones were picked up and cultured in Matrigel-coated 24-well plates and expanded for further certification. DNA sequencing was used to confirm the corrections of the HBB β41-42/β41-42 mutations. The HBA Westmead heterozygous point mutation was repaired based on the method used to correct HBB β41-42/β41-42 mutations mentioned above. The selection drug was G418 (100 µg/mL, Sigma, China).

### PCR analysis and sequencing of corrected clones

After drug selection, the positive clones were selected and further validated for genomic correction. The genomic DNA of these positive clones was extracted using TIANamp Genomic DNA Kit (Tiangen, China) according to the manufacture’s user manual. PCR was performed using 50-100 ng of genomic DNA templates and LA Taq (Takara, China). Designed primers included P1/P2, P3/P4, AEXON-F/BEXON-R. A 2.7-kb product of the 5’junction of a targeted integration was amplified using P1/P2. A 2-kb product or a 500 bp product was amplified by P3/P4 to identify whether homologous recombination occurred (Fig. 1A). A 600-bp product was amplified by AEXON-F/ BEXON-R and then was sequenced to identify whether the HBB mutations were corrected. Similarly, PF/PR was used to amplify a 2-kb product or a 500-bp product to determine whether random integration occurred (Fig. 1B). The primer pair HBA2 mut-F/R was used to amplify a 600-bp product containing the mutated region of HBA2. The PCR products were sequenced to identify whether the mutations were corrected. All primers sequences are listed in Table 1.

### Reverse transcription (RT) and quantitative PCR

Trizol (Invitrogen, China) was used to purify total RNA. Oligo dT (Takara, Japan) was used to reverse transcribe RNA into cDNA. Quantitative PCR (qPCR) was performed with SYBR green kit (Takara, Japan) on Agilent Technologies Stratagene Mx3000P. GAPDH was used for the reference gene. All data were measured in triplicate and repeated 3 times independently. Primer sequences used are presented in Table 1.

### Immunofluorescence

The cells were washed with PBS three times. Next, the cells were fixed with 4% paraformaldehyde for 30 min, followed by permeabilization of 0.3% Triton X-100. After cells were washed three times with PBS and blocked with 5% BSA, they were incubated with primary antibodies SOX2 (ab97959, Abcam, Cambridge, UK) or OCT4 (ab19857, Abcam, Cambridge, UK) overnight at 4 °C. Finally, the cells were washed three times with PBS. Relevant secondary antibodies were added for 1 h at room temperature, followed by the incubation with DAPI (Sigma, China) for 5 min in the dark.

### Teratoma formation analysis

βN/βN and αN/αN_corrected hiPSCs were cultured on Matrigel-coated 10-cm plates at 37 °C and 5% CO_2_. After reaching 80% confluency (about 1 × 10^6^ cells), these cells were digested accurate and resuspended in Matrigel (BD Biosciences, USA) and DMEM/F12 (1:2), and then injected 1 × 10^6^ cells subcutaneously into immune deficiency mice. Teratoma formation was evaluated upon sacrifice 8 weeks after the injection. It was dissected and fixed in 4% paraformaldehyde, followed by a dehydration series in ascending ethanol, clearing in xylene. Paraffin-embedded, formalin-fixed blocks were sectioned and stained with hematoxylin–eosin (HE).

### Extracorporeal induction of corrected iPSC hematopoietic differentiation

OP9 cells were mouse brain cap fibroblast cells. It can effectively induce hematopoietic differentiation of human pluripotent cells. OP9 cells were cultured in a 10-cm culture dish to a growth density of almost 90%. The β41-42/β41-42+ααWS/αα_iPS and βN/βN and αα/αα_corrected hiPSCs were scraped and collected after dispase (Invitrogen, China) digestion. They were then co-cultured with OP9 stromal cells for 10 days at 2.5 × 10^6^ cells per 10-cm culture dish in 20 mL co-culture medium containing α-MEM (Gibco, China), 10% fetal bovine serum (FBS, HyClone, USA) and 100 µM monothioglycerol (MTG, Sigma, China). The medium was replaced entirely on the second day and a half on day 4/6/8/10. Differentiated cells were collected on the 2nd, 4th, 6th, 8th, 10th, 12th day, respectively.

### Hematopoietic and erythroid colonies formation units assays

On day 10 after co-culture, 5 × 10^4^ cells of CD34^+^ hematopoietic cells were counted for the hematopoietic colony formation units (CFUs) assays by a direct CD34 Progenitor Cell Isolation Kit (Miltenyi Biotech, Germany). The cells were resuspended in 100 µL IMDM (Gibco, US) and 10% fetal bovine serum (FBS, HyClone, USA) added with 1-mL per dish of Metho Cult GF + H4435 semisolid medium (Stem cell Technologies, Canada) following the manufacturer’s instructions (Monroe, USA). Fourteen days later, erythroid colonies (Es) were counted and CFU-Es were collected to identify red blood cells. The level of β-globin protein was determined by flow cytometry (BD FACS Arial II, USA). PE Mouse Anti-Human CD71 (Cat.No.555537) was used to identify erythrocyte, and HBB antibody (Santa Cruz Biotechnology, sc-21757) was used to determine the level of β-globin.

### Whole-exome capture sequencing and gene-corrected off-target analysis

We performed whole-genome sequencing at × 100 coverage. All sequencing was performed using Illumina MGI V5 69 M (Illumina, San Diego, CA, USA), and exon capture was performed using Agilent SureSelect Technology (Agilent, Santa Clara, CA, USA). For sequence alignment, variant calling and annotation, these sequences were mapped to their locations with the human genome reference sequence (hg19; NCBI Build 37.1) using a Burrows-Wheeler Aligner (BWA) (v.0.5.9-r16). Single-nucleotide variations (SNVs) and indel variants were detected by a genome analysis tool (GATK v3.7).

### Statistical analysis

All statistical analyses were performed using SPSS 19.0 software to detect significant differences in measured variables among groups. A value of *P* < 0.05 was considered to indicate a statistically significant difference.

## Results

### hiPSCs derived from an α-and β-thalassemia carrier

The hiPSCs used in this study were generated from amniotic cells of a fetus with a homozygous mutation β41-42 (TCTT) deletion and a heterozygous mutation Westmead (*C* > *G*). We named this mutation β41-42/β41-42 and ααWS/αα. The β41-42 deletion was located in the second exon of the HBB gene and caused a frameshift. It generated a termination codon (TAG) in the position of the new 59th codon, which reduced the synthesis of the β chains of hemoglobin. The Westmead was located in the last exon CD122 (CAC > CAG) of the HBA2 gene.

### Detection of the cleavage activity of cas9/gRNA via a fluorescent reporter

To correct these mutations in *α*/*β* thalassemia, the oligos for β-sgRNA and α2-sgRNA were designed in 600 bp downstream of the last exon of HBB (Fig. 1A) and 200 bp downstream of the last exon of HBA2 (Fig. 1B), respectively. The complementary annealed sgRNA oligonucleotides were inserted into vector PX330. In order to evaluate the efficiency of gRNAs, GFP reporter and sgRNA plasmids were co-transfected into 293 T cells. After the targeting DNA was cut by Cas9, homologous recombination of the duplications occurred, resulting in the formation of a full-length GFP (Fig. 1C). The GFP signaling was detected 48 h after nucleofaction by FACS to assess the activity of sgRNA. The GFP expression in group transferred HBA2-sgRNA and HBA2-GFP reporter was increased more than 3 times compared to the group transfected HBA2-GFP reporter (Fig. 1D). The GFP expression in the group transferred HBB-sgRNA and HBB-GFP reporter was increased almost 3 times compared to the group transfected HBB-GFP reporter (Fig. 1E). These results demonstrated the efficient cleavage activity of HBB-sgRNA and HBA2-sgRNA.

### CRISPR-Cas9 technology correct α/β-thalassemia patient-derived hiPSC

**Fig. 2:**
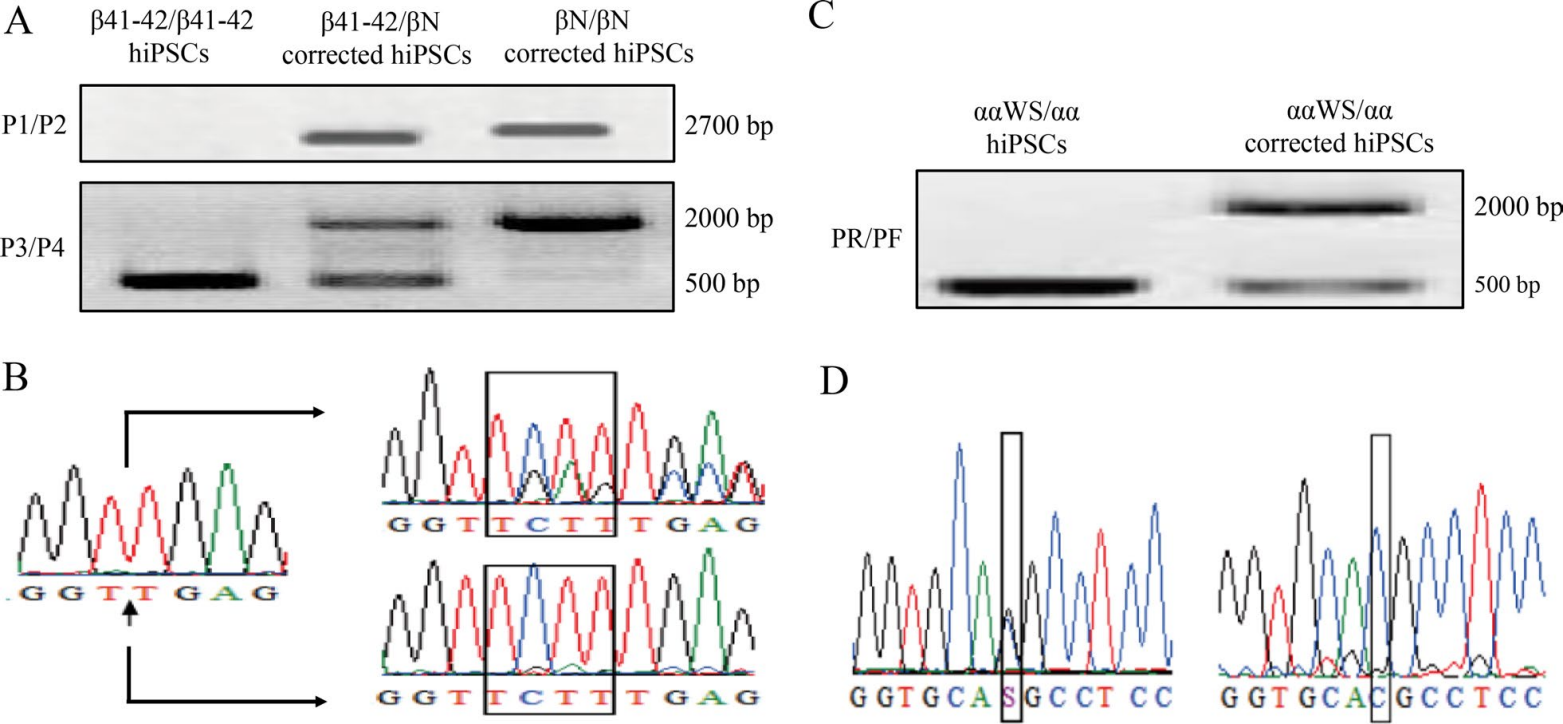
PCR and DNA sequence analysis of the corrected hiPSC clones. **A** After β41-42 homozygous mutation was completely corrected, the repaired clones showed two bands with different sizes in P1/P2 and P3/P4 amplification. **B** The black box indicates β41-42 deletion in the position of the new 59th codon. The sequencing results showed the β41-42 (-TCTT) heterozygous mutation before and after gene correction. **C** After the Westmead mutation was repaired, the repaired clones showed 500-bp and 2000-bp bands in PR/PF amplification. **D** The black box indicates the Westmead mutation was located in the last exon CD122 (CAC > CAG). Sequencing results showed the Westmead mutation (CAS → CAC) before and after gene correction. The P1/P2, P3/P4 and PR/PF were detected by PCR in different gels

We first corrected the mutations within the HBB gene. The HBB linearized donor and HBB-sgRNA were transfected into hiPSCs cells by electroporation. The positive clones were selected and transferred to Matrigel-coated 24-well plates after puromycin selection for further expansion and identification. We extracted the genomic DNA and amplified the desired genomic fragment. The repaired clones should give two bands of different sizes (Fig. 2A). We found that the repaired efficiency of β41-42/β41-42 homozygous mutations to β41-42/βN heterozygous mutations reached 90%, but homozygous correction (βN/βN) was only 1%. The PCR-identified positive clones were further confirmed through Sanger sequencing (Fig. 2B). The full-length gels are included in a supplementary information file.

To correct the mutations with HBA2, linearized donor and HBB-sgRNA were transfected into hiPSCs cells by electroporation. G418-resistant clones were picked and transferred into Matrigel-coated 24-well plates. We identified 5 of 34 clones as positive clones (Fig. 2C), with an efficiency of 14%. Sanger sequencing was performed for transcription of HBA2 restoration after genes correction (Fig. 2D).

### Gene-corrected α/β-thalassemia hiPSCs retain normal pluripotency

**Fig. 3:**
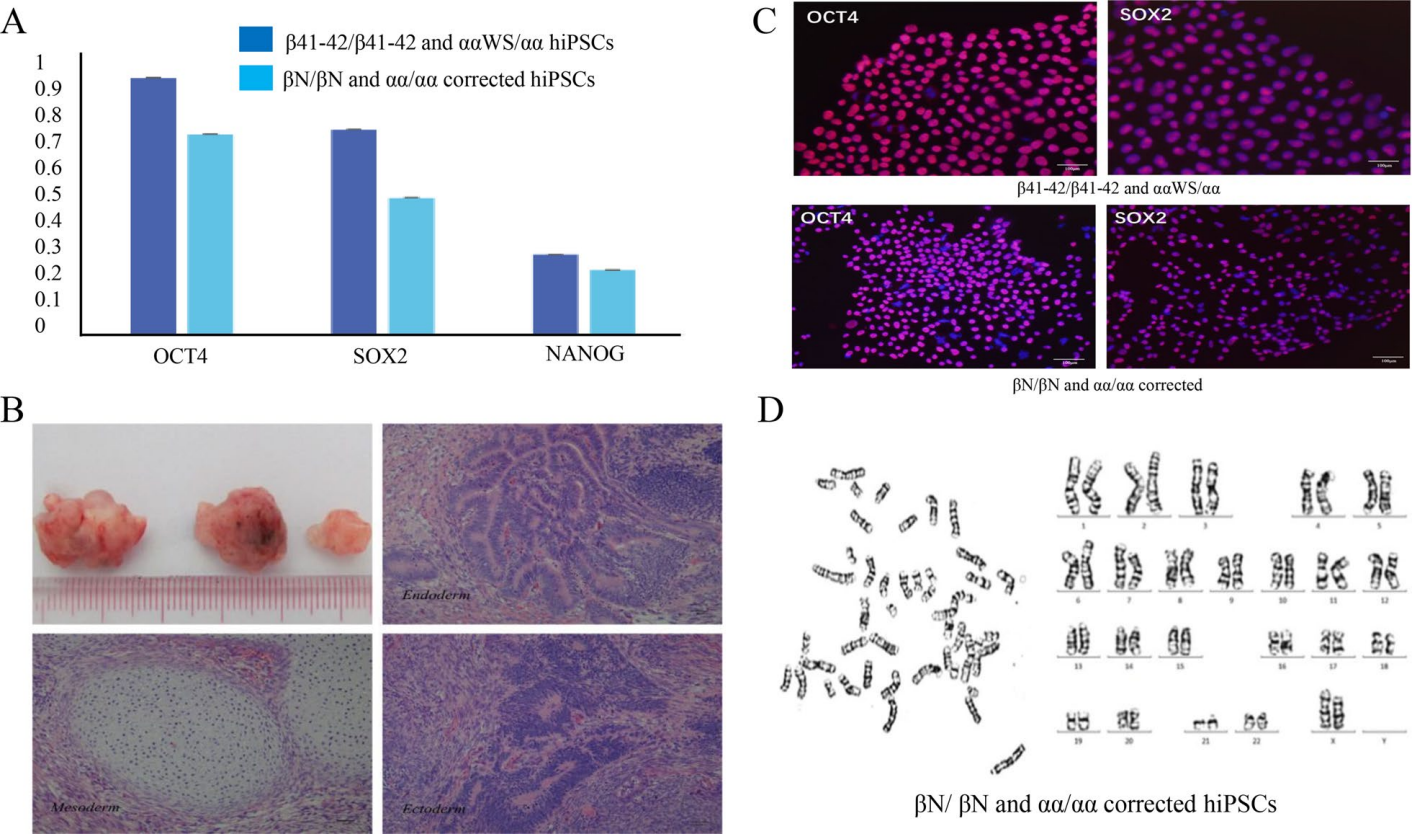
Characteristics of the corrected hiPSCs clones. **A** The expression level of pluripotent markers (OCT4, SOX2 and NANOG) in corrected hiPSC clones was analyzed by qRT-PCR. The gene-corrected clones maintained pluripotent genes. **B** Teratomas formation assay showed the differential ability of the corrected hiPSCs clones. **C** The expression of OCT4 and SOX2 in β41-42/β41-42 and αα/ααWS hiPSCs and βN/βN&αα/αα_corrected hiPSCs were detected by immunostaining. Scale bars = 100 µm. D, Karyotype analysis of the corrected hiPSCs clones showed that corrected hiPSCs retained normal female karyotype

To further identify whether the corrected hiPSCs still retained pluripotency, we evaluated the expression of the traditional pluripotent markers (OCT4, SOX2 and NANOG) using RT-qPCR. We found that all of these markers were well expressed in corrected hiPSCs (Fig. 3A). Also, these corrected hiPSCs maintained the stem cell morphology. In addition, these corrected cells were able to differentiate into three germ-layer lineages as revealed by teratomas formation assay (Fig. 3B). The expression of OCT4 and SOX2 was observed by immunostaining in corrected hiPSCs (Fig. 3C). Moreover, corrected hiPSCs still retained normal female karyotype (Fig. 3D).

### The gene-corrected off-target and exome sequencing analysis of gene-corrected hiPSCs

**Fig. 4:**
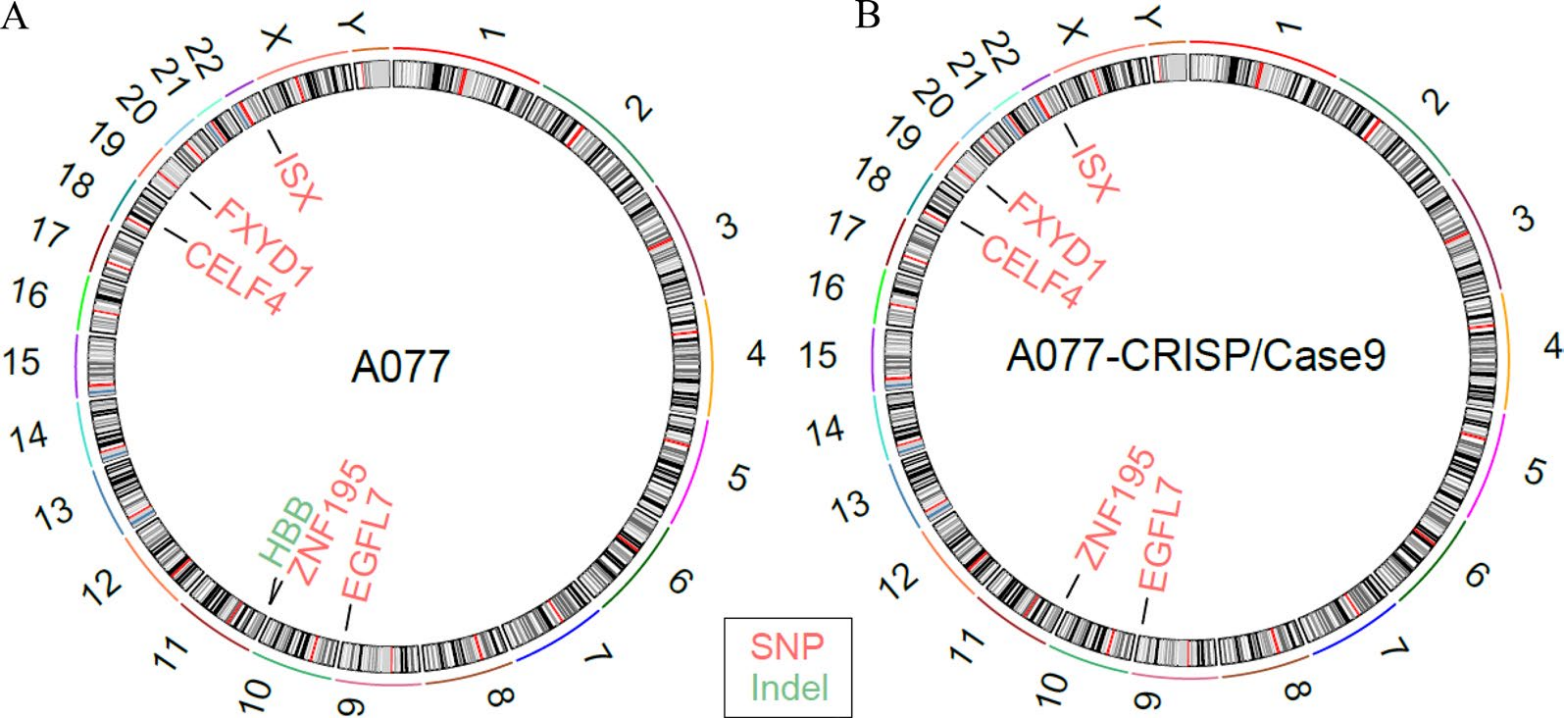
Whole-exome sequencing of the parental and gene-corrected hiPSCs. No obvious genome change was detected in parental and gene-corrected hiPSCs. Compared with the untargeted β-thal hiPSCs (**A**), the corrected hiPSCs (**B**) contain five the same single-nucleotide variations (SNVs), and one disappears indel than that formed from the hiPSCs without corrections

To discover the possible off-target events of the performance of CRISPR-Cas9, genomic DNA both from the before and after gene-targeted hiPSCs was examined by exome sequencing in comparison with the hg19 reference genome. Additionally, we predicted the potential off-target sites using the classical online software CRISPR Design. We found the top 7 off-target sites of HBA2 sgRNA and 30 off-target sites of HBB sgRNA, both of which within less than five mismatches. None of the mutations resides was located in potential off-target regions. Remarkably, compared to the uncorrected hiPSCs, the same 5 SNVs and a vanished indel in the corrected hiPSCs were detected in putative off-target sites. To sum up, these data strongly suggest that these mutations are not direct results of our off-target activities by Cas9 (Fig. 4).

### Differentiation of corrected hiPSCs into HSCs

**Fig. 5:**
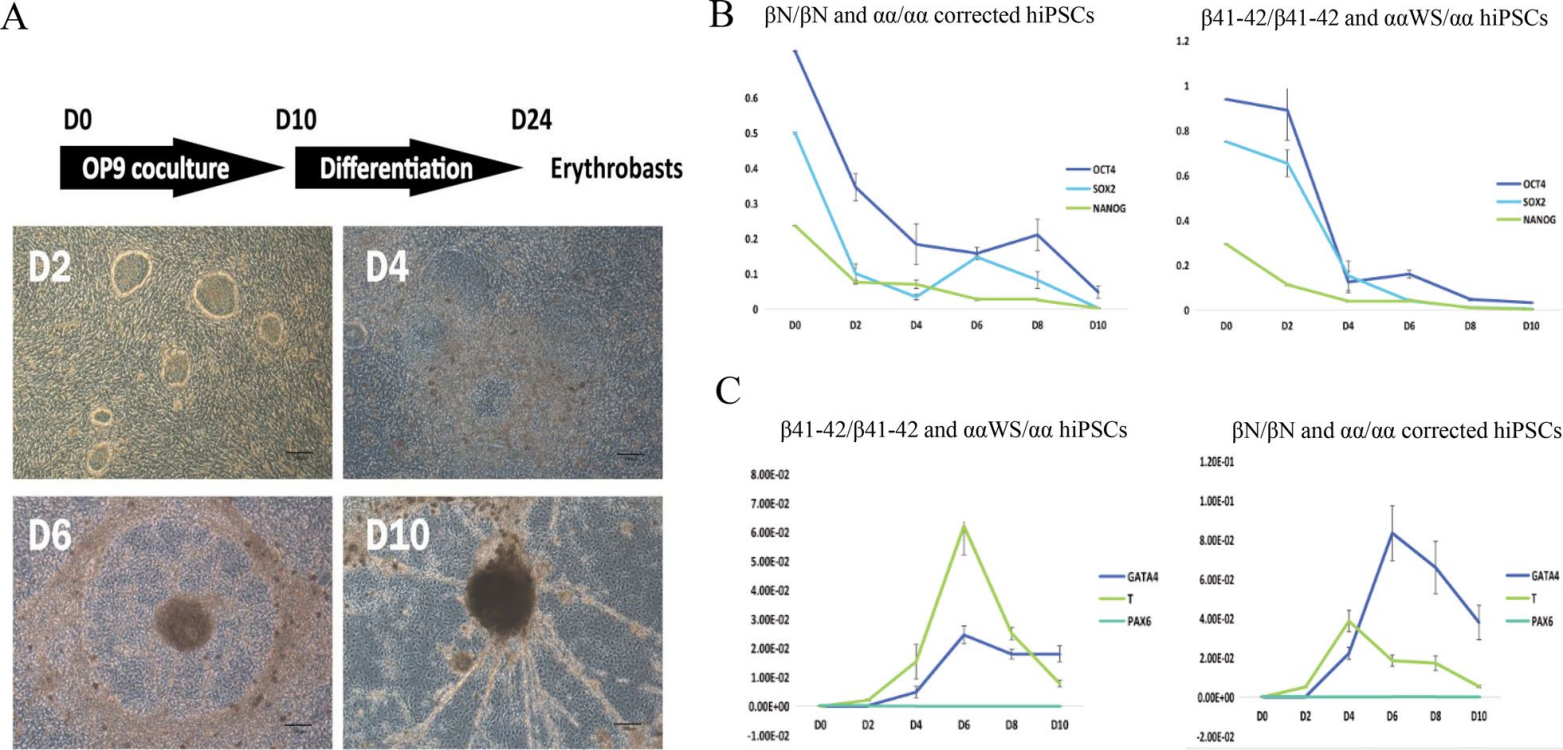
Hematopoietic differentiation of the corrected hiPSCs. **A** Morphological changes of the corrected hiPSCs cells during hematopoietic differentiation on day 2, day 4, day 6, day 10 of OP9 co-culture. Scale bars = 200 μm. **B** The expression level of pluripotent markers (OCT4, SOX2 and NANOG) in corrected hiPSCs clones was analyzed on day 0, day 2, day 4, day 6, day 8, day 10 of hematopoietic differentiation. Pluripotency genes OCT4, SOX2 and NANOG gradually decreased during hematopoietic differentiation, which is approaching 0. **C** The expression level of germ layer gene T in corrected hiPSCs clones was analyzed on day 0, day 2, day 4, day 6, day 8, day 10 of hematopoietic differentiation. During the hematopoietic differentiation process, the germ layer gene T expression peak appeared earlier in repaired hiPSCs than that no corrected hiPSCs

Hematopoietic differentiation experiment and colony-forming assay were performed to examine the hematopoietic function of the corrected hiPSCs. OP9 cells were mouse brain cap fibroblast cells. It can effectively induce hematopoietic differentiation of human pluripotent cells without other cytokines. OP9 cells were co-cultured with the corrected or uncorrected hiPSCs (Fig. 5A). The expression of OCT4, SOX2, NANOG, GATA4 and PAX6 was examined by RT-qPCR. The results revealed that the expression of pluripotency genes, OCT4, SOX2 and NANOG, gradually decreased, and the mesoderm gene T was expressed earlier in the corrected hiPSCs compared to uncorrected hiPSCs during the differentiation process (Fig. 5B and C).

**Fig. 6:**
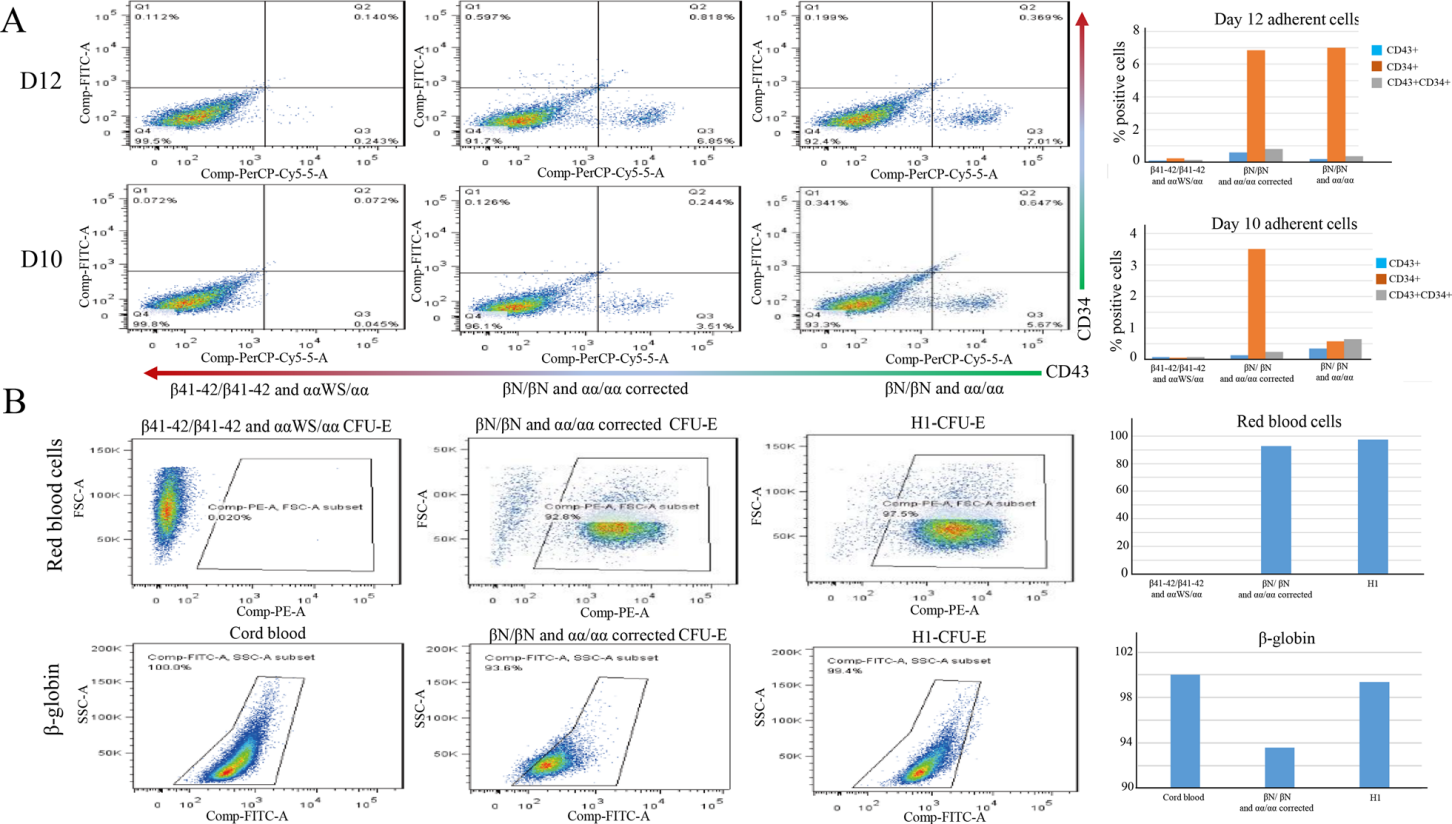
Flow cytometry analyses of the surface marker in hematopoietic stem cells. **A** Flow cytometry revealed the results of anti-human CD34 + /CD43 + in the corrected hiPSCs. Flow cytometry analysis of CRISPR/Cas9-corrected hiPSCs showed significantly higher hematopoietic differentiation. **B** Flow cytometry revealed the results of anti-human CD71 and anti-HBB in the corrected hiPSCs CFU-E flow analysis results showed the expression of β-globin in repaired hiPSCs, which is no significant difference between cord blood and H1. Moreover, no red blood cells were detected in the unrepaired hiPSCs

**Fig. 7:**
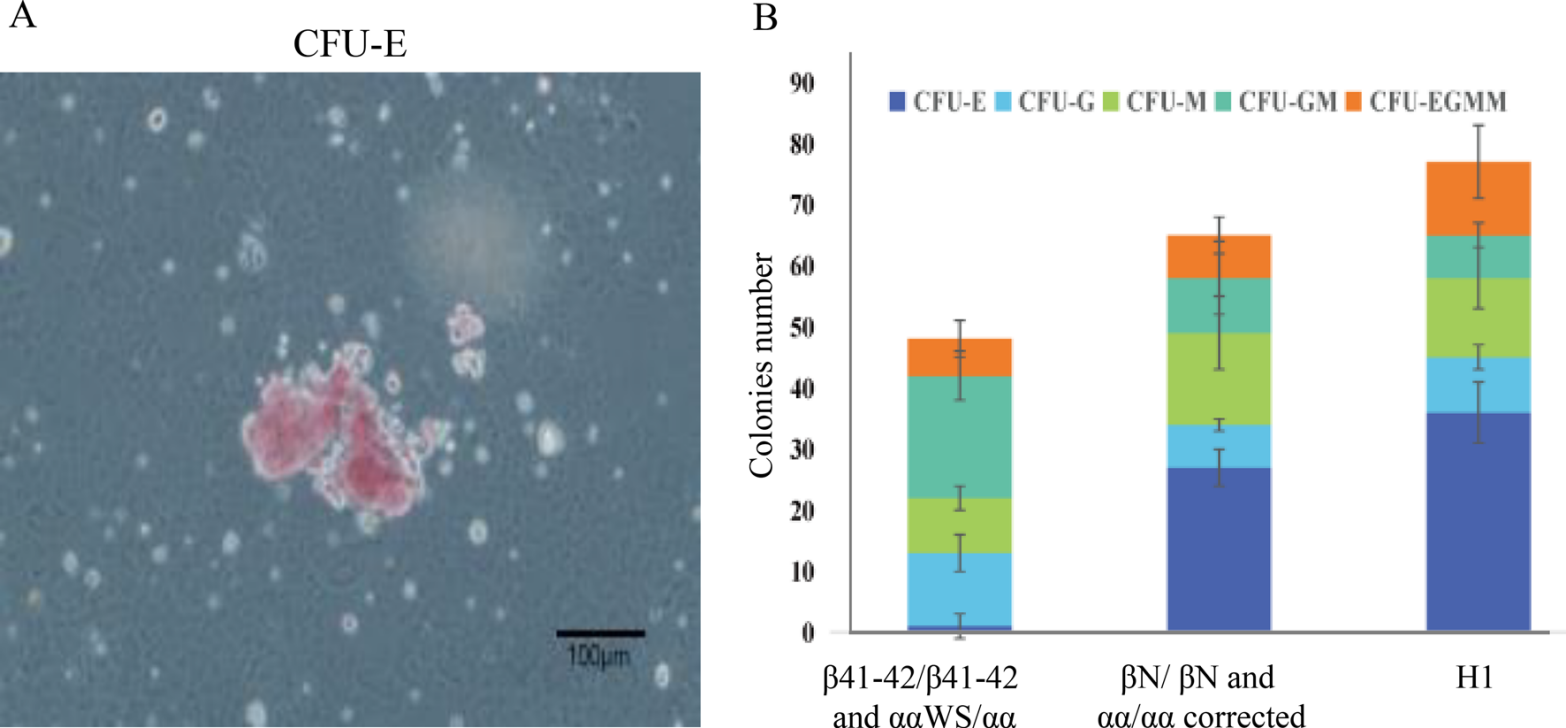
CFU-E colony formation. **A** Representative image of CFU-E. Scale bars = 200 µm. **B** The number of CFU-E formed by hiPSCs after the repair was significantly higher than that no corrected hiPSCs

CD34^+^ and CD43^+^ are hallmark surface markers of HSCs in humans. The red blood lineage clones were picked up and examined by flow cytometry. Flow cytometry analysis of CRISPR/Cas9-corrected hiPSCs showed significantly higher hematopoietic differentiation by analyzing the number of CD34^+^/CD43^+^ cells (Fig. 6A). However, compared with the cord blood or H1 cells, the gene-repaired hiPSCs cell lines did not show any significant differences in the expression levels of β-globin (Fig. 6B). Interestingly, the number of CFU-E from the corrected hiPSCs was significantly higher than those from uncorrected hiPSCs (Fig. 7), indicating these cells could differentiate into different blood lineages.

## Discussion

Hb Constant Spring (Hb CS, c.427 *T* > *C*), Hb Quong Sze (Hb QS, HBA2: c.377 *T* > *C*) and Hb Westmead (Hb WS, HBA2: c.369 *C* > *G*) are three common mutations causing nondeletional α-thal in the Chinese population [17]. A nondeletional α-thal usually is associated with mild clinical symptoms [18]. However, it is reported that nondeletional α-thal in combination with β0-thal causes β-thalassemia intermedia (β-TI) presented with a significant variety of clinical presentations [19]. Due to its complex clinical presentation, early valid intervention for α- and β-thalassemia is not frequently applied until complications of iron overload or other thalassemia-related complications occur later [20]. At present, allogeneic hematopoietic stem cells and bone marrow transplantation are the only possible curative therapy for thalassemia [21]. However, immune rejection and lack of HLA-matched donors hamper their clinical implementation [22].

In recent years, a growing number of studies have reported using the CRISPR-Cas9 technique to correct the mutation from allele of the HBB gene by homology-directed repair with a single-stranded DNA oligonucleotide template [23]. As the third generation of engineered endonuclease, CRISPR-Cas9 is proving to be an efficient and customizable alternative to other existing genome editing tools [24]. For example, the HBB gene CD41/42(-CTTT) mutation has been successfully repaired by the CRISPR-Cas9 technique [25]. The HBA2 gene with a Hb-CS mutation has been successfully corrected in a patient-specific hiPSCs [26]. In the present study, we reported a patient with an HBB homozygous deletion mutation (CD41-42) and an HBA heterozygous point mutation (Hb WS). We designed the gRNAs recognized the β41-42 mutation sequence on the HBB gene and the long sequence for the Hb WS mutations on the HBA2 gene. After transfecting the long linearized donor plasmids and Cas9 gRNA into hiPSCs, the corrected clones were selected by antibiotic selection. Since off-target mutagenesis is a primary safety concern of CRISPR-Cas9 therapy, the potential off-target sites were confirmed by DNA sequencing. Our results indicated that the monoalleleic correction efficiency reached 90%. The efficiency of biallelic correction was 14%, much higher than the correction efficiency of HbH-CS from a previous report with a 6.3% for monoallelic correction and 16.7% for biallelic correction [27].

Recent reports have shown the success of genetic correction of an HBB or HBA2 gene in thalassemia-specific hiPSCs using the CRISPR-Cas9 technique [28]. hiPSCs have certain advantages over other stem cell types in human disease treatment because they are derived from adult somatic cells and not embryos [29]. It can be continuously expanded in vitro and is amenable to genetic manipulation [30]. hiPSCs also are not associated with the ethical dilemmas surrounding the use of embryonic stem cells [31]. Our study demonstrated that transplantation of the corrected autologous patient-derived hiPSCs could be potential treatment strategies for preventing thalassemia. A gene-corrected hiPSCs lineage could provide HLA-matched cell type for all pathological tissues and organs of interest [32]. Human pluripotent stem cells can self-renew and differentiate into hematopoietic cells [33]. We found that the expression of pluripotency-related genes and transient early mesoderm markers in corrected hiPSCs was gradually decreased during the differentiation process. CD34 and CD43 are the well-known surface markers of HSCs [34]. CD34^+^ is used to identify hematopoietic stem cells with multilineage potential [35]. We further found that the number of CD34^+^/CD43^+^ cells from the gene-corrected group was significantly higher than those from the uncorrected group, indicating that gene-corrected hiPSCs have multilineage differentiation potential.

## Conclusions

In summary, our study provides a successful strategy to repair two disease-causing mutations in human mutant hiPSCs. This could be employed as a universal approach in the future correction of the HBB and HBA gene in hiPSCs derived from β41-42/β41-42 and ααWS/αα-thalassemia patients. The knowledge and protocols obtained from our study will apply to the genetic correction of patient-specific hiPSCs with other genetic disorders.

## Data Availability

The datasets used and/or analyzed during the current study are available from the corresponding author on reasonable request.

## Abbreviations

BWA: Burrows-Wheeler aligner
CFUs: Colony formation units
CRISPR: Clustered regularly interspaced short palindromic repeat sequences
FACS: Fluorescence-activated cell sorter
FBS: Fetal bovine serum
GFP: Green fluorescent protein
GAPDH: Glyceraldehyde-3-phosphate dehydrogenase
HBA2: Human hemoglobin alpha 2
HBB: Human β-globin
HE: Hematoxylin/eosin
hiPSCs: Human-induced pluripotent stem cells
HSCs: Hematopoietic stem cell
MSX1: Msh homeobox 1
OCT4: Organic cation/carnitine transporter4
PAX6: Paired box 6
PBS: Phosphate-buffered saline
RT-qPCR: Reverse transcription-quantitative PCR
SOX2: SRY-box transcription factor 2
SNVs: Single-nucleotide variations

## References

[1] Cao A, Kan YW (2013). The prevention of thalassemia. Cold Spring Harb Perspect Med.

[2] Shang X, Xu X (2017). Update in the genetics of thalassemia: what clinicians need to know. Best Pract Res Clin Obstet Gynaecol.

[3] De Sanctis V, Kattamis C, Canatan D, Soliman AT, Elsedfy H, Karimi M (2017). Beta-thalassemia distribution in the old world: an ancient disease seen from a historical standpoint. Mediterr J Hematol Infect Dis.

[4] He S, Qin Q, Yi S, Wei Y, Lin L, Chen S (2017). Prevalence and genetic analysis of alpha- and beta-thalassemia in Baise region, a multi-ethnic region in southern China. Gene.

[5] Wang Z, Sun W, Chen H, Zhang Y, Wang F, Chen H (2021). Prevalence and molecular spectrum of alpha- and beta-globin gene mutations in Hainan, China. Int J Hematol.

[6] Sabath DE (2017). Molecular diagnosis of thalassemias and hemoglobinopathies: an ACLPS critical review. Am J Clin Pathol.

[7] Taher AT, Weatherall DJ, Cappellini MD (2018). Thalassaemia. Lancet.

[8] Zhang W, Cai WW, Zhou WP, Li HP, Li L, Yan W (2008). Evidence of gene conversion in the evolutionary process of the codon 41/42 (-CTTT) mutation causing beta-thalassemia in southern China. J Mol Evol.

[9] Colombatti R, Sainati L, Trevisanuto D (2016). Anemia and transfusion in the neonate. Semin Fetal Neonatal Med.

[10] Swart JF, Delemarre EM, van Wijk F, Boelens JJ, Kuball J, van Laar JM (2017). Haematopoietic stem cell transplantation for autoimmune diseases. Nat Rev Rheumatol.

[11] Amjad F, Fatima T, Fayyaz T, Khan MA, Qadeer MI (2020). Novel genetic therapeutic approaches for modulating the severity of beta-thalassemia (review). Biomed Rep.

[12] Li T, Luo C, Zhang J, Wei L, Sun W, Xie Q (2021). Efficacy and safety of mesenchymal stem cells co-infusion in allogeneic hematopoietic stem cell transplantation: a systematic review and meta-analysis. Stem Cell Res Ther.

[13] Naldini L (2015). Gene therapy returns to centre stage. Nature.

[14] Clement K, Hsu JY, Canver MC, Joung JK, Pinello L (2020). Technologies and computational analysis strategies for CRISPR applications. Mol Cell.

[15] Germini D, Tsfasman T, Zakharova VV, Sjakste N, Lipinski M, Vassetzky Y (2018). A comparison of techniques to evaluate the effectiveness of genome editing. Trends Biotechnol.

[16] Long P, Wang Z, Yang H, Liu Z, Wu B, Zhong G, Chen J, Sun C, Wang F, Zhou Y, Sun F, Li Q, Ma Y (2020). Generation of nine iPSC lines (HNMUi002-A, HNMUi003-A, HNMUi004-A, HNMUi005-A, HNMUi006-A, HNMUi007-A, HNMUi008-A, HNMUi009-A, HNMUi010-A) from three Chinese families with thalassemia. Stem Cell Res.

[17] Huang Q, Wang X, Tang N, Yan T, Chen P, Li Q (2017). Simultaneous genotyping of alpha-thalassemia deletional and nondeletional mutations by real-time PCR-based multicolor melting curve analysis. J Mol Diagn.

[18] Farashi S, Harteveld CL (2018). Molecular basis of alpha-thalassemia. Blood Cells Mol Dis.

[19] Fucharoen S, Fucharoen G (2012). Hb H disease with various beta hemoglobinopathies: molecular, hematological and diagnostic aspects. Hemoglobin.

[20] Asadov C, Alimirzoeva Z, Mammadova T, Aliyeva G, Gafarova S, Mammadov J (2018). Beta-thalassemia intermedia: a comprehensive overview and novel approaches. Int J Hematol.

[21] Srivastava A, Shaji RV (2017). Cure for thalassemia major: from allogeneic hematopoietic stem cell transplantation to gene therapy. Haematologica.

[22] Locatelli F, Merli P, Strocchio L (2016). Transplantation for thalassemia major: alternative donors. Curr Opin Hematol.

[23] Pattabhi S, Lotti SN, Berger MP, Singh S, Lux CT, Jacoby K (2019). In vivo outcome of homology-directed repair at the HBB gene in HSC using alternative donor template delivery methods. Mol Ther Nucleic Acids.

[24] Jacinto FV, Link W, Ferreira BI (2020). CRISPR/Cas9-mediated genome editing: from basic research to translational medicine. J Cell Mol Med.

[25] Niu X, He W, Song B, Ou Z, Fan D, Chen Y (2016). Combining single strand oligodeoxynucleotides and CRISPR/Cas9 to correct gene mutations in beta-thalassemia-induced pluripotent stem cells. J Biol Chem.

[26] Yingjun X, Yuhuan X, Yuchang C, Dongzhi L, Ding W, Bing S (2019). CRISPR/Cas9 gene correction of HbH-CS thalassemia-induced pluripotent stem cells. Ann Hematol.

[27] Razzouk S (2018). CRISPR-Cas9: a cornerstone for the evolution of precision medicine. Ann Hum Genet.

[28] Pavani G, Fabiano A, Laurent M, Amor F, Cantelli E, Chalumeau A (2021). Correction of beta-thalassemia by CRISPR/Cas9 editing of the alpha-globin locus in human hematopoietic stem cells. Blood Adv.

[29] Moradi S, Mahdizadeh H, Šarić T, Kim J, Harati J, Shahsavarani H (2019). Research and therapy with induced pluripotent stem cells (hiPSCs): social, legal, and ethical considerations. Stem Cell Res Ther.

[30] Sugimoto N, Eto K (2021). Generation and manipulation of human hiPSC-derived platelets. Cell Mol Life Sci.

[31] Volarevic V, Markovic BS, Gazdic M, Volarevic A, Jovicic N, Arsenijevic N (2018). Ethical and safety issues of stem cell-based therapy. Int J Med Sci.

[32] Wahlster L, Daley GQ (2016). Progress towards generation of human haematopoietic stem cells. Nat Cell Biol.

[33] Slukvin II (2013). Hematopoietic specification from human pluripotent stem cells: current advances and challenges toward de novo generation of hematopoietic stem cells. Blood.

[34] Zhu Y, Wang T, Gu J, Huang K, Zhang T, Zhang Z (2020). Characterization and generation of human definitive multipotent hematopoietic stem/progenitor cells. Cell Discov.

[35] Sonoda Y (2021). Human CD34-negative hematopoietic stem cells: The current understanding of their biological nature. Exp Hematol.

